# Uptake of Biosimilar Infliximab in the UK, France, Japan, and Korea: Budget Savings or Market Expansion Across Countries?

**DOI:** 10.3389/fphar.2020.00970

**Published:** 2020-07-09

**Authors:** Yujeong Kim, Hye-Young Kwon, Brian Godman, Evelien Moorkens, Steven Simoens, SeungJin Bae

**Affiliations:** ^1^ College of Pharmacy, Ewha Woman’s University, Seoul, South Korea; ^2^ Strathclyde Institute of Pharmacy and Biomedical Sciences, University of Strathclyde, Glasgow, United Kingdom; ^3^ College of Pharmacy, Seoul National University, Seoul, South Korea; ^4^ Division of Clinical Pharmacology, Karolinska Institutet, Karolinska University Hospital, Stockholm, Sweden; ^5^ Department of Public Health Pharmacy and Management, School of Pharmacy, Sefako Makgatho Health Sciences University, Garankuwa, South Africa; ^6^ Department of Pharmaceutical and Pharmacological Sciences, KU Leuven, Leuven, Belgium

**Keywords:** biosimilar, market penetration, infliximab, market dynamics, cross national comparisons, Korea

## Abstract

**Objective:**

To compare the market dynamics of biosimilar infliximab among four Organization for Economic Cooperation and Development (OECD) countries (UK, France, Japan, and Korea) where supply-side and demand-side policies varied greatly, given high and growing expenditure on biological medicines to treat immunological diseases across countries.

**Methods:**

A quarterly dataset covering October 2012 to March 2018 was constructed from the MIDAS-IQVIA International database. The sales value (in USD) and volume (in standard units) of originator infliximab and biosimilar products and their relative price in each country were compared.

**Results:**

With the introduction of biosimilars, the sales value of infliximab increased approximately 2.5 times in Korea, whereas it only slightly increased (1.2 times for France and the UK) or decreased (0.9 for Japan) in other countries. While stable market size dynamics were observed in the other countries, an escalating market size, attributable to the increase in originator infliximab, was observed in Korea. In the UK and France, which have implemented demand-side policies, the sales volume of originator infliximab appreciably decreased after the entry of biosimilar infliximab while that of biosimilars increased; however, in Korea, which has supply-side policies based on price-linking with few demand-side policies, the volume of originator infliximab actually increased by 70% alongside a very limited increase in biosimilar infliximab. The lowest price ratio between biosimilar and originator infliximab was found in Japan, at 68%. In France and Korea, the ex-factory prices of biosimilar infliximab were 99 and 95%, respectively, of the originator infliximab price. In the UK, the ex-factory price of biosimilar infliximab started at 87% of that of originator infliximab and then decreased to 80% as the market matured. However, actual price differences might differ.

**Conclusion:**

The uptake of biosimilar infliximab varied greatly, and in contrast to the UK, France, and Japan, the introduction of biosimilar infliximab resulted in market expansion in Korea, which might be explained by a lack of demand-side policies in Korea. Both supply- and demand-side measures are necessary for health authorities to achieve desired savings from the availability of biosimilars.

## Introduction

Biological medicines are among the most expensive medicines on the market, with average costs at approximately 22 times the cost of nonbiological drugs (Richardson, 2013). The global market for biotechnology products accounted for 25% ($208 billion) of the total global pharmaceutical market of US$825 billion in 2017, and biotechnology products within the top 100 products based on annual expenditures have grown from just 2% in 2010 to 49% in 2017 (Malik, 2018). Accordingly, the impact of these products on overall health expenditures has rapidly increased (Elsevier Drug Information, 2017; GaBi, 2017; Gleeson et al., 2019).

Recently, a number of standard biological medicines have faced patent expiration, sparking increased interest in biosimilars (Blackstone and Joseph, 2013; Mestre-Ferrandiz et al., 2016; GaBi, 2017; Moorkens et al., 2017; Santos et al., 2019). Adalimumab and omalizumab lost exclusivity rights in 2018, and trastuzumab is scheduled to lose its patent between 2014 and 2019 in a number of countries (Pivot et al., 2018; Thill, 2019), followed by bevacizumab and ranibizumab (GaBi, 2017). Accordingly, a number of biosimilars are expected to actively enter the market soon, helping to further address the budgetary concerns that have accompanied the increasing usage of high-cost biological medicines (Moorkens et al., 2017). Consequently, a better understanding of market dynamics upon the entry of biosimilars is needed to guide future activities.

According to Lubloy (2014), factors affecting new medicine uptake are likely to have a variety of underlying economic mechanisms. He classified these factors into macro-, meso-, and micro-level characteristics. Macro-level includes policies such as patent expiration, regulatory requirements, pricing and reimbursement policies, and others. Meso-level characteristics are prescribing characteristics of doctors, marketing efforts of pharmaceutical companies, interpersonal communication among doctors, drug attributes, and characteristics of patients, and micro-level characteristics include sociodemographic and professional characteristics of medical professionals. All of them influence the uptake of new medicines (Lubloy, 2014). “Biosimilar” is a regulatory term used to define a biological medicine highly similar to another already approved biological medicine (a “reference medicine”) (European Medicines Agency, 2019; US Food and Drug Administration, 2019). Biosimilars differ from generic medicines in that they have highly similar, but not the same, active ingredients compared to the reference products (Richardson, 2013; US Food and Drug Administration, 2019). Several countries have implemented policies to encourage the utilization of biosimilars including educational initiatives, physician incentives, and (limited) pharmacist substitution (Moorkens et al., 2017; Godman et al., 2019). In this context, health insurers and health authorities expect potential financial savings from biosimilars; however, savings are likely to be lower in reality with initially higher priced biosimilars (Haustein et al., 2003; Richardson, 2013; Mulcahy et al., 2017; Smeeding et al., 2019). The potential for cost savings from biosimilars has been seen in a number of studies (Haustein et al., 2003; Razanskaite et al., 2017), and this will continue. The magnitude of cost savings though may be different from that seen with generics due to several clinical concerns, including safety and immunogenicity issues, necessitating comparative clinical studies as opposed to just bioavailability studies. However, a number of studies have now suggested that earlier clinical concerns are less of an issue in reality (Jorgensen et al., 2017; Komaki et al., 2017; Stebbing et al., 2017; Vergara-Dangond et al., 2017; Cantini and Benucci, 2018), yet each country has different policies toward biosimilars. As a result, there is a need to review both supply- and demand-side policies across countries for biosimilars to enable countries to learn from each other given the level of potential savings that can be achieved with these high expenditure medicines. We are aware that the use of biological medicines varies considerably across Europe with currently appreciably lower use among Central and Eastern European countries versus Western European countries (Putrik et al., 2014; Kostic et al., 2017; Baumgart et al., 2019). However, in this paper we wanted to concentrate on both supply- and demand-side measures where biologicals are already used and reimbursed for these conditions to help with future policies. No doubt current restrictions and issues of affordability surrounding the use of biological medicines for immunological diseases will be eased by the availability of appreciably lower cost biosimilars; however, this is outside the scope of this study. Thus, this study aimed to explore the uptake of biological medicines after the entry of biosimilars and focus on the macro-level factors affecting biosimilar uptake including key pricing and usage-enhancing policies of each country (Lubloy, 2014; Rémuzat et al., 2017a; Rémuzat et al., 2017b).

### Key Pricing (Supply-Side) and Usage-Enhancing (Demand-Side) Policies on Biosimilars in Selected Countries

As mentioned, biosimilar policies vary across countries. The United Kingdom, France, Japan, and South Korea provide a mixture of countries in terms of funding of health care (insurance or taxation), medicine co-payment levels, geography, and both supply- and demand-side measures for biosimilars.

#### The United Kingdom

As biosimilars are typically the least expensive treatment option following tendering, there have been a number of ongoing strategies in the United Kingdom (UK) to accelerate their use in hospitals (e.g., infliximab) and the community *via* home care services (Razanskaite et al., 2017). For instance, Scotland produced guidance to enhance the use of biosimilars in cases where a biological medicine is being considered to help conserve resources as well as allay concerns about their possible effectiveness and safety versus those of the originator in 2015 (Health Improvement Scotland (NHS Scotland), 2015), which was updated in 2018 (Health Improvement Scotland (NHS Scotland), 2018). To further enhance the prescribing of biosimilars, National Helath Services (NHS) Scotland in 2016 highlighted successful switching programs. The push to switch to biosimilars was assisted by the British Society of Rheumatology announcing its support for biosimilars in February 2015 (NHS Scotland, 2016). In addition, biosimilar use is regularly tracked by NHS Scotland as part of national therapeutic indicators (NHS Scotland, 2017). These various regional and national activities can help address concerns among healthcare professionals, especially regarding switching (Aladul et al., 2018; Aladul et al., 2019), with NHS England currently aiming for 90% of new patients to be prescribed the best-value biological medicine within 3 months of the launch of a biosimilar as well as actively encouraging switching (NHS England, 2017a) to meet the goal of an 80% biosimilar prescription rate within one year (Davio, 2018). NHS England has also invested in many educational activities (NHS England, 2015; NHS England, 2017b; NHS England, 2019a) and closely monitors local adoption of biosimilars through regional teams that facilitate implementation of national policy measures (NHS England, 2019b). The use of gainsharing agreements, where part of the savings are shared between commissioners and providers, also provide an important incentive for biosimilar adoption (Syrop, 2017). In addition, there has been further instigation of competitive pricing involving multiple companies to avoid the formation of monopolies (Davio, 2018). These combined policies for biosimilars have resulted in significant estimated savings for the UK. The estimated savings for infliximab were GB£99.4 million in 2017, for etanercept GB£60.3 million, and for rituximab GB£50.4 million, with cumulative savings estimated at US$275 million (Davio, 2018).

#### France

The uptake of biosimilars in France has been supported at the national level. The French National Medicines Agency (ANSM, Agence Nationale de Sécurité du Médicament et des Produits de Santé) initially recommended against switching the prescription of patients already treated with a biologic (ANSM, 2013). However, ANSM changed its position in May 2016 because of the positive real-world evidence available on biosimilars (ANSM, 2016). In October 2017, a new ministerial instruction (Ministère des Solidarités et de la Santé, 2018) stated that more than 70% of the treatment initiation in ambulatory patients must be performed with biosimilars where available and that switches must be encouraged. At the same time, ANSM created a reference list of biosimilar products, which included infliximab, to facilitate switching between originators and biosimilars (ANSM, 2019). In the National Health Strategy 2018–2022, the targeted biosimilar uptake level was raised to 80% in ambulatory patients (GaBi, 2018). With respect to biosimilar substitution, the French situation has evolved over time. While the 2014 Social Security Financing law in theory provided a legal basis for pharmacy substitution under certain conditions for treatment-naïve patients, as well as for some existing patients, to ensure continuity of biosimilar treatment, the 2020 Social Security Financing law has abolished this possibility of pharmacy substitution (Ordre national des pharmaciens, 2020). However, as an implementing decree has never been enacted, pharmacy substitution was never allowed in practice (GaBi, 2014). In the retail setting, the price of originator biologics is reduced by 20%, and the price of biosimilars is expected to be 40% below the originator’s initial price. Price revision for both products every 18–24 months is based on the penetration rate of biosimilars (price cut between 5 and 15%) (EY Advisory and Consulting Co, 2018; TLV, 2018; World Health Organization, 2018). Biosimilars that are used in the hospital setting, such as infliximab, are included in tender processes on a hospital level. List prices and hospital reimbursement of the originator and biosimilar are the same, and any savings resulting from discounts given in the tender process are equally shared between the hospital and payer (Simon-Kucher and Partners. Bonn, 2016). In 2018, hospitals that have engaged in a contract to improve prescribing quality and efficiency could receive additional remuneration depending on their prescription rate of biosimilar etanercept and biosimilar insulin for ambulatory patients (Ministère des Solidarités et de la Santé, 2018).

#### Japan

The Ministry for Health Labor and Welfare (MHLW) issued the biosimilar guidelines for market approval of biosimilars in 2009, which is based on the EU regulatory process (GaBI Online, 2016).

In terms of pricing of biosimilars, Japan strictly regulates the price of biosimilars yet has few usage-enhancing policies. Specifically, biosimilars are priced 30% lower than originators based on a price-link policy when they are listed at the National Health Insurance program, yet usage-enhancing policies such as International nonpropriety name (INN) prescribing, biosimilar substitution, and prescription guidelines that encourage biosimilar uptake are currently not implemented. Furthermore, no clinical treatment guidelines regarding biosimilars appears to have been issued in Japan (Hara et al., 2019). In addition, a recent study reported that patients and pharmacists are not familiar with biosimilars, and 43% of oncologists expressed concerns regarding insufficient clinical evidence with biosimilars, resulting in unfamiliarity with biosimilars and limited utilization in practice (Tanabe et al., 2016). Consequently, it seems that physicians are not familiar with biosimilars and face neither incentive nor regulation to increase biosimilar uptake. In view of this Japan can be classified as a country with strong supply-side policy (price regulation), yet few demand-side (usage-enhancing) policies.

#### South Korea

The Korean Ministry of Food, Drug and Safety (MFDS) issued biosimilar product regulatory guidelines including biosimilar approval, selection of reference drugs, and quality, non-clinical and clinical testing of biosimilars in 2010 (Jung, 2015). This guideline is similar to the European, Japanese, and WHO guidelines in terms of the scope and data requirements for authorization.

Korea also has a supply-side policy with few demand-side measures. Biosimilars were priced approximately 30% lower than originators; however, this discount has been reduced to just 20% as part of a Reform Plan for Reimbursement Prices of Biopharmaceuticals and Global Innovative Pharmaceuticals in 2016 (Jung, 2015; EY Advisory and Consulting Co, 2018). The tendering process is used only in public hospitals (Ministry of Government Legislation, 2020a; Ministry of Government Legislation, 2020b; Ministry of Government Legislation, 2020c), which constitute fewer than 6% of all hospitals in Korea (OECD Health Database, 2017). Policies to enhance biosimilar uptake, such as substitution, INN prescribing, reference pricing, prescription guidelines or monitoring of prescription patterns, are not currently in place, meaning that only one supply-side measure, namely, a price-link policy, is in effect and that other biosimilar uptake policies are lacking.

#### Summary

In summary, the selected OECD countries (except the UK) have similar pricing policies yet very different biosimilar usage-enhancing policies (**Table 1**). The UK has the most aggressive usage-enhancing policies (targeting prescriptions of biosimilars for 90% of new patients within three months) and tendering processes, with the policies in France falling in between (with switching and prescribing guidance), and Korea and Japan currently have no usage-enhancing policies in place.

**Table 1:** Biosimilar policies in four OECD countries, 2012–2018.

Countries	Pricing policies			Usage-enhancing policies				
	Price fixing to the price of originator		Tendering	Substitution	Switching	Prescription guidelines	Monitoring of prescription patterns
	First biosimilar	Follow-on					
UK	Free pricing PPRS rules apply	No specific pricing regulation	**√**		**√**	**√**	**√**
France	Below 60%^a^	Info not available	**√**		**√**	**√**	
Japan^c^	70%: 10% of Premium can be granted based on the types of the clinical trials	60% of the first biosimilar after 10th biosimilar being listed					
Korea (Ministry of Health and Welfare, 2019)	70~80%	None	**√** ^b^				

a At least 40% lower than the originator’s initial price (TLV, 2018).

b Only public hospitals [6% of the number of hospitals (OECD Health Database, 2017)] are engaged in tendering (Ministry of Government Legislation, 2020a; Ministry of Government Legislation, 2020b; Ministry of Government Legislation, 2020c).

c Ministry of Health, Labor, and Welfare. Update of Drug Pricing System in Japan, (Yomoto, 2017).

Despite the differences in biosimilar usage-enhancing policies, few studies have compared and examined the impact of those policies and tried to explain their implications in terms of market dynamics, volume evolution, and cost savings. We sought to explore these market dynamics (in terms of volume and expenditure) after the entry of biosimilar infliximab in the UK, France, Japan, and Korea.

## Methods

### Data Sources

We constructed a quarterly dataset, extracted from the MIDAS-IQVIA International database, for October 2012 to March 2018 (22 quarters) pertaining to the sales value (ex-manufacturer sales in current US dollars converted from local currencies) and volume (referred to as standard units (SU) of 100 mg/vial) for both originator and biosimilar infliximab products. SU represents the number of standard “dose” units sold and has been used in previous studies using MIDAS-IQVIA data to observe utilization patterns (Magazzini et al., 2004; Duggan et al., 2014; Van Boeckel et al., 2014).

IQVIA data has been used in multiple studies including cross-national and national studies including those for biological medicines especially where it has been difficult to obtain utilization and expenditure data from national data sources (Danzon et al., 2011; Diao et al., 2019; Schieber et al., 2019; Moorkens et al., 2019). Data from IQVIA was considered the optimal source for this study involving medicines dispensed in both hospitals and ambulatory care.

### Selection of the Biosimilars for the Study and the Countries

For international comparison, common active substances for which biosimilar versions were available in the four selected countries were screened. Biosimilars based on the genetic recombinant technology (i.e. monoclonal antibodies) of five substances (adalimumab, etanercept, infliximab, rituximab, and trastuzumab) have been approved in each country. Of these, we selected biosimilars for which the sales value could be tracked for more than two years. As a result, only infliximab [L04AB02 (WHO, 2019)] was selected for the study since its biosimilar version was first approved in July 2012 in Korea, in Europe in March 2013 by the EMA (but allowed on the market only since 2015), and July 2014 in Japan. The other four molecules were not selected due to the insufficient data coverage for the four compared countries during the study period.

For the purpose of international comparison, we selected OECD countries where biosimilar infliximab was available for the given time period and sufficiently observable for the comparison. Accordingly, the market dynamics of biosimilar infliximab in Korea were compared with those in France, the UK and Japan, as these countries exemplify distinct pricing, reimbursement, and demand-side policies to include in the analysis. In this way, market trends can be discussed in light of varying national policy measures, and potential influences of these measures can be explored.

### Outcome Variables

To understand the market dynamics of biosimilars compared to the originator, we focused on analyzing the following three factors: (1) sales value (in USD), (2) sales volume, and (3) price competition.

First, the sales value of infliximab was observed to explore to what extent the sales of infliximab changed after the entry of biosimilar infliximab, and these findings can also help predict potential savings to the healthcare system. Accordingly, quarterly sales of infliximab after the entry of the biosimilar were calculated as a ratio with reference to the infliximab sales value at the biosimilar entry point (Q=0). In addition, sales value ratios for both originator and biosimilar infliximab were separately calculated to show the market changes after the entry of competitive biosimilars.

For utilization, the sales volume share of biosimilar infliximab was used to proxy the uptake of biosimilar infliximab over that of its counterpart. This share was calculated by the sales volume of biosimilars (in SU) divided by the sales volume of both the biosimilar and originator infliximab (in SU). Additionally, the sales volume ratio of both originator and biosimilar infliximab was separately calculated by dividing the sales volume for each quarter with reference to the sales volume at the time when biosimilar infliximab was introduced (Q=0).

The price of the biosimilar versus that of its originator was calculated at each point in time. Prices were calculated by dividing the sales value by the sales volume. Consequently, the price represents the price of the standard unit (SU), which is equivalent to the ex-factory price. We are aware that the price we used may not be the actual purchasing price, since the actual price is confidential in countries like France and the UK. However, the use of ex-factory prices is the second best alternative and the same methodology has been applied in previous studies comparing international prices (Danzon and Furukawa, 2006; Kanavos et al., 2013). The trend of the price ratio shows the price level of the biosimilars compared to the originator, along with the price competition between the two infliximabs over time.

To increase the comparability of the market dynamics in each country, we benchmarked the time points of the observations from the specific time of entry of biosimilar infliximab in each country (Q=0).

## Results

### Overview of Infliximab Uptake by Sales

**Figure 1:**
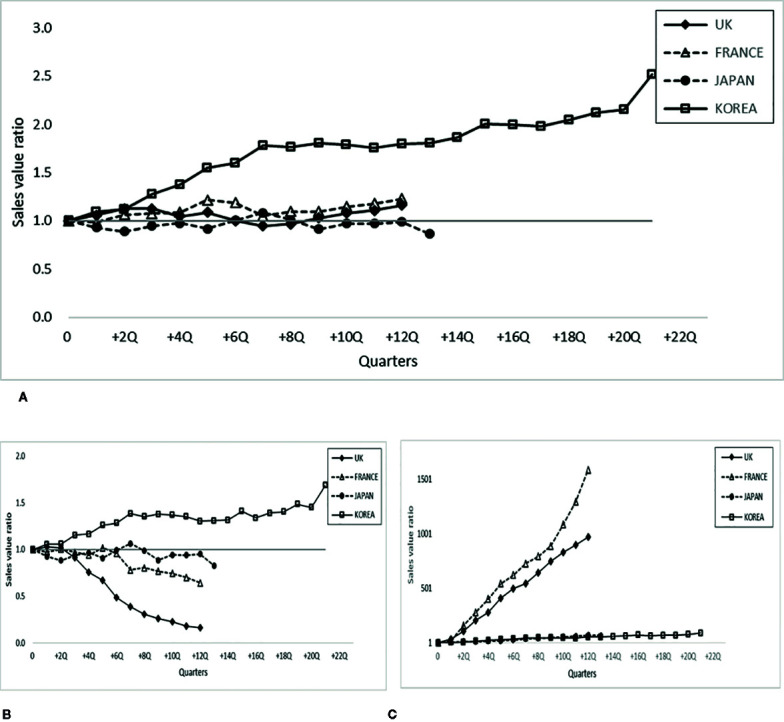
Sales value ratio of infliximab after the entry of biosimilar infliximab in 4 countries. **(A)** Sales value ratio of infliximab (originator and biosimilar) at the time shown in the quarter (numerator) and at the time of biosimilar entry (Q=0, denominator, reference). **(B)** Sales value ratio of originator infliximab at the time shown in the quarter (numerator) and at the time of biosimilar entry (Q=0, denominator, reference). **(C)** Sales value ratio of biosimilar infliximab at the time shown in the quarter (numerator) and at the time of biosimilar entry (Q=0, denominator, reference).

**Figure 1** shows the infliximab market trend with the introduction of infliximab biosimilar(s) in the four countries and their relative sales values. The infliximab market size in terms of expenditure based on ex-factory prices has not changed significantly in all countries except Korea, where a substantial increase was observed (**Figure 1A**).Specifically, the infliximab sales value in Korea increased by approximately 2.5 times compared with that the time when the biosimilar was first launched whereas other countries showed a minimal or steady trend overtime (20% in the UK and France, -10% in Japan after 12 quarters).

**Figure 1B** shows that the sales value (expenditure) of originator infliximab decreased in the countries where the market size decreased (UK, France, and Japan), whereas the opposite trend was observed in Korea, where the sales value of originator infliximab increased by approximately 1.7 times with the introduction of the biosimilar. Among those countries where the market size of originator infliximab decreased, the markets in the UK and France plummeted (approximately 80 and 40%, respectively), and Japan slightly decreased (20%).

**Figure 1C** shows that the sales value of the biosimilar substantially increased in France (1,587.7 times) and the UK (972.6 times), whereas a more limited increase was observed in Japan (67.7 times) and Korea (89.6 times). Overall, the stable size dynamics in some markets in **Figure 1A**, namely, the UK, France, and Japan, also experienced a decrease in originator infliximab sales value, whereas an increase in the expenditure of originator infliximab was noted in Korea, where an escalating market size was observed. Interestingly, a decrease in originator infliximab accompanied an increase in biosimilar infliximab in the UK and France, which was not the case in Japan.

### Biosimilar Infliximab Market Share by Sales Volume

**Figure 2:**
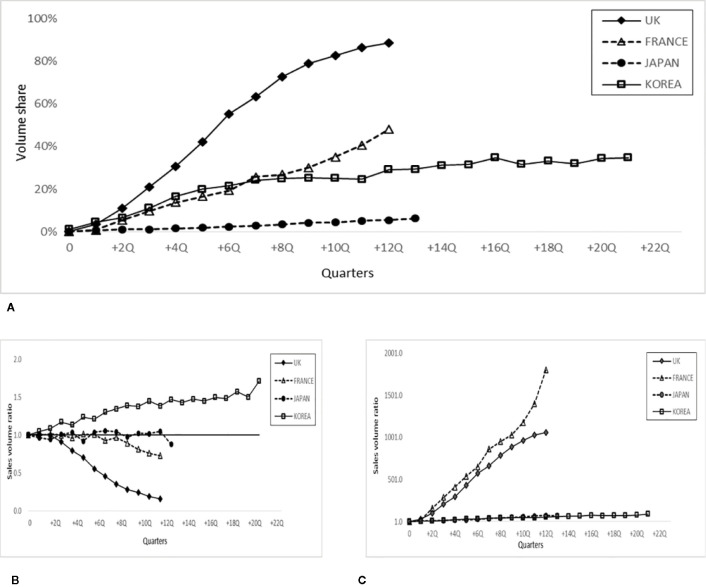
Share of biosimilar infliximab and sales volume ratio after the entry of biosimilar infliximab in 4 countries. **(A)** Evolution of the biosimilar infliximab share over the total sales volume for each quarter. **(B)** Sales volume ratio of originator infliximab at the time shown in the quarter (numerator) and at the time of biosimilar entry (Q=0, denominator reference). **(C)** Sales volume ratio of biosimilar infliximab at the time shown in the quarter (numerator) and at the time of biosimilar entry (Q=0, denominator, reference).

To examine whether the market expansion was associated with the use of originator infliximab or its biosimilar(s), **Figure 2A** shows the share of biosimilar infliximab based on total sales volume during the study period. The share of infliximab biosimilar(s) reached approximately 89% in the UK, followed by France (48%), and Korea (35%), whereas it was only 6% in Japan, based on the values for quarter one of 2018 (the last time point in the data set). **Figures 2B, C** show the sales volume ratio of the originator infliximab and its biosimilar(s), respectively. In the UK, the relative volume of originator infliximab appreciably decreased by 80% (**Figure 2B**), yet the volume of the biosimilar increased by 1,058 times compared to the quarter when biosimilar infliximab was introduced (**Figure 2C**), based on the last observed value.

A similar trend was observed in France, yet in Korea, the volume of originator infliximab actually increased by 70% during this period (**Figure 2B**) alongside a very limited increase in biosimilar infliximab (**Figure 2C**), suggesting that the increased market size observed in **Figure 1** is associated with increased utilization of originator infliximab. In Japan, the utilization of originator infliximab and its biosimilar counterparts was rather stable (**Figure 2B**): the use of the former slightly decreased and of the latter increased marginally.

### Relative Ex-Factory Price of Biosimilar Infliximab Compared With the Originator

**Figure 3:**
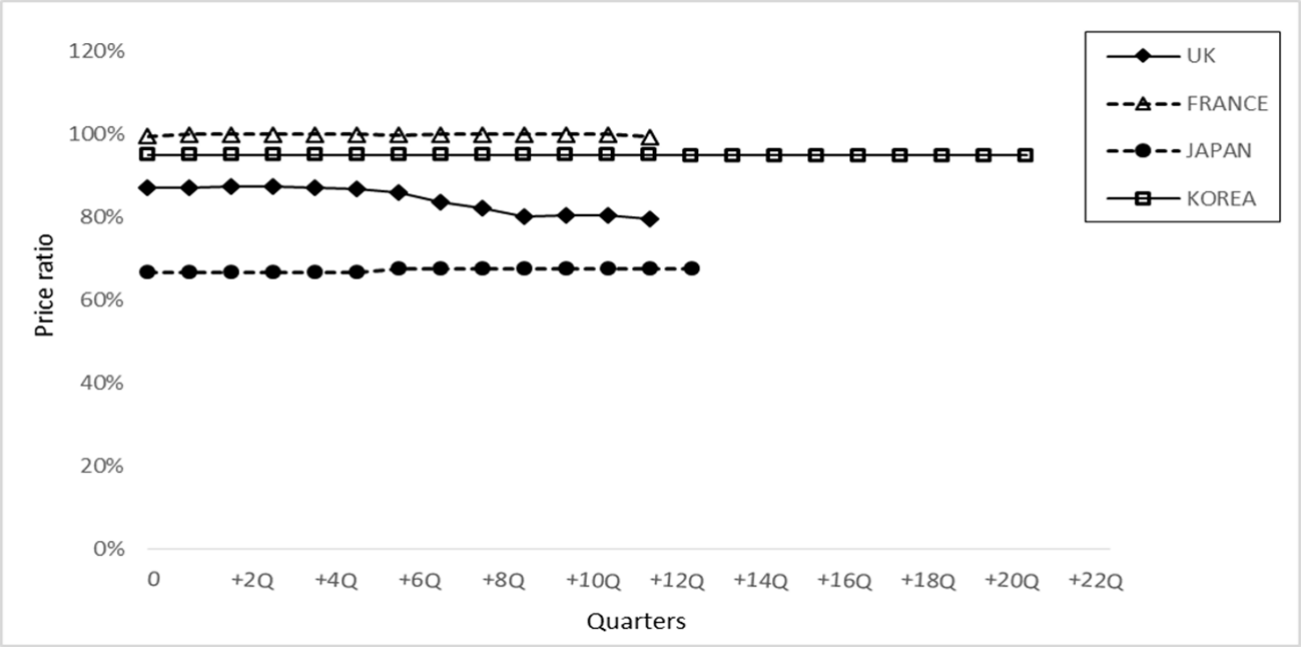
Price ratio of biosimilar infliximab in 4 countries. The price ratio is defined as the price of biosimilar infliximab (numerator) with reference to the price of originator infliximab (denominator) in each period.

**Figure 3** shows the relative price level between originator infliximab and its biosimilar counterparts, which indicates the potential financial savings with the introduction of infliximab biosimilar(s). The lowest price ratio was found in Japan, where the price of biosimilar infliximab was approximately 68% of that of the originator and remained consistent during the study period. In France and Korea, the prices of biosimilar infliximab were 99 and 95%, respectively, of the originator infliximab price, and the price level remained stable. In the UK, the price of biosimilar infliximab started at 87% of the originator infliximab price and then decreased as the market matured (80%).

## Discussion

This study explored the market dynamics after the entry of biosimilar infliximab among four countries including the UK, France, Japan, and Korea, using 22 quarters of MIDAS-IQVIA observational data up to March 2018 focusing on the macro-level factors such as pricing and usage enhancing policies in each country.

Our study emphasized the importance of demand‐side policies in biosimilar market penetration. With the introduction of biosimilar infliximab, increased use of both the originator infliximab and the biosimilars were observed in Korea, which contrasted with other countries, suggesting that the introduction of biosimilars can actually increase the financial burden without demand side policies depending on the level of price reductions actually achieved. Our analysis showed that the share of biosimilar infliximab rapidly increased and its originator counterpart decreased in the UK and France. The UK has demonstrated the largest biosimilar uptake, with the originator infliximab market share plummeting and biosimilar infliximab market share soaring simultaneously.

Although substitution is not allowed, the UK has various usage-enhancing policies to encourage biosimilar market penetration, such as setting prescription guidelines that alleviate concerns over the safety and effectiveness of biosimilars as well as prescribing targets. Considering that unfamiliarity and concerns over interchangeability have been reported as the main obstacles to biosimilar uptake (Aladul et al., 2018), targeting health care providers or patients and increasing familiarity could enhance the uptake of biosimilars. We are aware that the price of biosimilars is typically determined based on tendering in the UK. However, our analysis showed that the biosimilars were priced approximately 33% lower than the original biologic, which is typically lower than countries with price regulations (France and Korea). This may also be an underestimate, especially if a number of contracts are confidential. It has been reported that biosimilar uptake is closely related to demand-side policies, which target health care providers and/or patients (Rémuzat et al., 2017b; Vogler and Schneider, 2017). The UK case demonstrates the importance of demand-side policies, with the introduction of biosimilars rapidly resulting in cost savings by replacing the originator with biosimilars. This can be seen with adalimumab in the UK where a mixture of aggressive tendering coupled with demand-side measures is expected to result in savings of approximately GB£300 million (approximately $386 million) from expenditure levels of GB£400 million-per-year (approximately $514 million) prior to biosimilars (Davio, 2018). We have seen other examples in the UK that the combination of aggressive supply-side measures coupled with demand-side measures can achieve considerable efficiencies whilst improving care. In the case of lipid-lowering medicines measures to achieve very low prices for generics, coupled with demand-side measures to increase their prescribing and at higher doses to reduce morbidity and mortality from cardiovascular disease, resulted in a 50% reduction in overall expenditure between 2001 and 2015 despite a 412% increase in utilization (Leporowski et al., 2018).

France showed a similar trend, with the introduction of biosimilar infliximab resulting in substantial uptake of the biosimilar at the expense of its originator. On the demand side, policies supporting interchangeability and switching target health care providers were introduced to increase the market penetration of biosimilars. In addition, hospital tenders, which might not always have a biosimilar as a winner, and local management play an important role.

The relative price level of biosimilars in Japan is lowest among the four countries, at approximately 33% lower than the originator (Ministry of Health, Labour and Welfare, 2018). Interestingly, despite the relative price advantage of the biosimilar, the uptake of biosimilar infliximab was negligible in Japan, which might be due to the relative unfamiliarity with biosimilars among healthcare providers and patients in Japan (Hara et al., 2019) coupled with the lack of automatic substitution or prescribing targets. Overall, Japan demonstrated very strong supply-side policies based on price-link policies, yet the lack of demand-side policies may well have resulted in poor uptake of biosimilars. This needs to be addressed if Japan is to benefit from the availability of lower-cost biosimilars.

Unlike the other three countries, Korea presented an interesting and unique phenomenon. The sales value of the biosimilar and its originator increased 2.5 times by the end of the study period compared with the time when the biosimilar was first launched. Market expansion was observed for both the originator and the biosimilars, suggesting that the introduction of the biosimilar did not come close to achieving financial savings. Considering that generic drugs or biosimilars are typically encouraged to achieve financial savings, the introduction of biosimilars in this case resulted in the opposite outcome. This phenomenon is consistent with the findings of Kwon and Godman (2016), who demonstrated that the introduction of generics actually resulted in a market expansion effect in Korea after the market entry of generic statins, which can be explained by the increase in the number of patients receiving prescriptions for generic atorvastatin enhanced by increased promotional activities by generic manufacturers (Lee and Lee, 2013; Kwon and Godman, 2016). It is interesting to note that our findings are consistent with those on statins, which are used in outpatient settings, where the target population is not fully defined and supplier-induced demand is more likely to occur, as most of the services are reimbursed based on a fee-for-service model. However, infliximab, unlike statins, is mostly indicated for hospitalized patients (those affected by, e.g., Crohn’s disease, ulcerative colitis, rheumatoid arthritis), and inelastic demand is assumed. Consequently, it is surprising that similar to generic statins, the introduction of biosimilar infliximab actually resulted in market expansion. The lack of demand-side policies (usage-enhancing policies) and no marked price differentiation between originators and generics in Korea to encourage the use of generics, also impacted on their use (Kwon and Godman, 2016). These market expansions of biosimilar and originator infliximab may be partly explained by supplier-induced demand in Korea (Lee and Lee, 2013; Kwon and Godman, 2016). In addition, lowering prices means that more patients can be treated for the same budget, which is important where there are restrictions on the use of biological medicines exist due to issues of affordability. In addition, in countries where there are high co-payments for medicines such as Central and Eastern European countries, the availability of low cost biosimilars is expected to appreciably increase the use of biological medicines for immunological diseases from current low bases (Kostic et al., 2017; Baumgart et al., 2019). However, future studies on the meso- and microlevels, such as the characteristics of prescribers and patients, are needed to determine the detailed reasons for the market expansion specifically shown in Korea, and we will follow up on this in future research.

Japan, while similar to Korea in terms of lacking demand-side measures, demonstrated a different dynamic from that of Korea. No market expansion was observed in Japan, but strong price regulations were imposed after the entry of biosimilar infliximab. Sales values and volumes of the originator decreased in Japan following the introduction of biosimilars, while they increased in Korea. This outcome should be further researched in light of increasing discussions regarding the rational use of medicines.

There are several limitations in our study. First, the study period is rather short in some countries, and only four countries were selected, which might prevent us from comparing market dynamics with sufficient rigor. However, biosimilars have only been recently introduced with various launch dates across countries, and biosimilar infliximab is a common molecule that has been introduced in many countries with a relatively long observation period. In addition, the four countries in our study represent different health care systems with different supply-side and demand-side policies. Second, our results are based on only one biosimilar, and thus the findings might not be generalizable to other biosimilars. However, considering that biosimilars including adalimumab, etanercept, and rituximab are mostly indicated for severe diseases and likely to be used by similar medical specialties, we assume that a similar trend would be observed if we increased the sample size. Further studies are needed, especially as mentioned in light of the initiatives by AbbVie to appreciably lower the price of Humira^®^ (originator adalimumab) across Europe since the loss of exclusivity in October 2018 to help reduce competition (Sagonowsky, 2019). Lastly, the price we used in this study may not reflect the true price because of the nature of MIDAS-IQVIA data in that it is based on sales quantity collected and produces the projected sales value by multiplying the unit sold by a price at the manufacturer level (IQVIA, 2009). “Real” prices are not available for the UK and France since the actual prices are negotiated at the hospital level or *via* decentralized purchasing structures, which are kept confidential. Given that tender prices are usually effective in hospital settings, true prices are likely to be much more dynamic, and we were unable to capture these prices in this study. Despite these limitations, this study firstly attempted to explore the market dynamics of biosimilar infliximab in Korea compared with three other countries, i.e., the UK, France and Japan, in terms of supply-side and demand-side measures, and secondly to suggest some policy implications by reviewing the supply- and demand-side measures in place in each country. It is salient that Korea is the only country that shows counterintuitive market dynamics, which are an undesirable signal for its National Health Insurance system. Future studies should include a more in-depth analysis of how regional and local practices contribute to originator and biosimilar market dynamics as more biosimilars are launched. More countries could also be included to extend our findings and build on this research to make a global comparison.

## Conclusion

The market uptake of biosimilar infliximab varied greatly, and the introduction of biosimilar infliximab resulted in a market expansion in Korea, in contrast with those in the UK, France, and Japan. Increased use of the biosimilars as well as the originator was observed in Korea, which might be explained by a lack of demand-side policies. Government agencies should give greater consideration to demand-side alongside supply-side policies to enhance the cost savings achieved by the introduction of biosimilars in the Korean context.

## Data Availability Statement

The datasets generated for this study will not be made publicly available: IMS-MIDAS can be purchased and accessed at IQVIA.

## Ethics Statement

Ethical approval was not required as only market information was used for this study. The data were hosted and managed by IQVIA for the commercial use and purchased for this study.

## Author Contributions

H-YK and SB conceived the study design and YK analyzed the data. YK, H-YK, SB, BG, EM, and SS developed the draft of manuscript. All authors contributed to the article and approved the submitted version.

## Conflict of Interest

SS is one of the founders of the KU Leuven Fund on Market Analysis of Biologics and Biosimilars following Loss of Exclusivity (MABEL). SS was involved in a stakeholder roundtable on biologics and biosimilars sponsored by Amgen, Pfizer, and MSD and has participated in an advisory board meeting for Pfizer. SS has contributed to studies on biologics and biosimilars for Hospira, Celltrion, Mundipharma, Pfizer, and Amgen.

The remaining authors declare that the research was conducted in the absence of any commercial or financial relationships that could be construed as a potential conflict of interest.

## References

[B1] Aladul M. I. Fitzpatrick R. W. Chapman S. R. (2018). Healthcare professionals’ perceptions and perspectives on biosimilar medicines and the barriers and facilitators to their prescribing in UK: a qualitative study. BMJ Open 8, e023603. 10.1136/bmjopen-2018-023603 PMC6252648 30455389

[B2] Aladul M. I. Fitzpatrick R. W. Chapman S. R. (2019). Differences in UK healthcare professionals’ knowledge, attitude and practice towards infliximab and insulin glargine biosimilars. Int. J. Pharm. Pract. 27, 214–217. 10.1111/ijpp.12485 30160324

[B3] ANSM (2013). ANSM report: les médicaments biosimilaires: etat des lieux (ANSM). Available at: http://ansm.sante.fr/S-informer/Points-d-information-Points-dinformation/L-ANSM-publie-un-etat-des-lieux-sur-les-medicamentsbiosimilaires-Point-d-information (Accessed 22 Dec. 2020).

[B4] ANSM (2016). ANSM report: etat des lieux sur les médicaments biosimilaires (ANSM). Available at: http://ansm.sante.fr/var/ansm_site/storage/original/application/c35f47c89146b71421a275be7911a250.pdf (Accessed May 2019).

[B5] ANSM (2019). Reference list of similar biological groups (ANSM). Available at: https://ansm.sante.fr/Activites/Medicaments-biosimilaires/Les-medicaments-biosimilaires/(offset) (Accessed 22 Dec. 2019).

[B6] Baumgart D. C. Misery L. Naeyaert S. Taylor P. C. (2019). Biological Therapies in Immune-Mediated Inflammatory Diseases: Can Biosimilars Reduce Access Inequities? Front. Pharmacol. 10, 279. 10.3389/fphar.2019.00279 30983996 PMC6447826

[B7] Blackstone E. A. Joseph P. F. (2013). The economics of biosimilars. Am. Health Drug Benefits 6, 469–478. 24991376 PMC4031732

[B8] Cantini F. Benucci M. (2018). Focus on biosimilar etanercept-bioequivalence and interchangeability. Biologics 12, 87–95. 10.2147/BTT.S126854 30214149 PMC6121755

[B9] Danzon P. M. Furukawa M. F. (2006). Prices and availability of biopharmaceuticals: an international comparison. Health Affairs 25 (5), 1353–1362. 10.1377/hlthaff.25.5.1353 16966733

[B10] Danzon P. M. Furukawa M. F. (2011). "Cross-National Evidence on Generic Pharmaceuticals: Pharmacy vs. Physician-Driven Markets," NBER Working Papers 17226 (National Bureau of Economic Research, Inc.).

[B11] Davio K. (2018). After biosmilar deals, UK spending on Adalimumab will drop by 75% (The Center for Biosimilars). Available at: https://www.centerforbiosimilars.com/news/after-biosimilar-deals-uk-spending-on-adalimumab-will-drop-by-75 Accessed 13 Jan 2020.

[B12] Diao Y. Qian J. Liu Y. Zhou Y. Wang Y. Ma H. (2019). How government insurance coverage changed the utilization and affordability of expensive targeted anti-cancer medicines in China: an interrupted time-series study. J. Glob. Health 9 (2). 10.7189/jogh.09.020702 PMC6815654 31673344

[B13] Duggan M. Garthwaite C. Goyal A. (2016). The market impacts of pharmaceutical product patents in developing countries: Evidence from India. Am. Econ. Rev. 106 (1), 99–135. 10.1257/aer.20141301 31178595

[B14] Elsevier Drug Information (2017). Managing the costs of specialty drugs. https://www.elsevier.com/__data/assets/pdf_file/0009/473787/4A_Managing-the-Costs-of-Specialty-Drugs.pdf. Accessed June 2020.

[B15] European Medicines Agency (2019). Biosimilar medicines: Overview (European Medicines Agency). Available at: https://www.ema.europa.eu/en/human-regulatory/overview/biosimilar-medicines-overview (Accessed 11 Feb 2019).

[B16] EY Advisory and Consulting Co (2018). Global biosimilar policy comparison. (EY Advisory and Consulting Co.). Available at: https://www.eyadvisory.co.jp/services/documents/pdf/global-biosimilar-policy-comparison-report_final.pdf (Accessed 21 May 2019).

[B17] GaBI Online (2016). Japanese guidelines for biosimilars (Mol, Belgium: Pro Pharma Communications International). Available from: www.gabionline.net/Guidelines/Japanese-guidelines-for-biosimilars Accessed 7 Feb 2020.

[B18] GaBi (2014). France to allow biosimilars substitution. Available at: http://gabionline.net/Policies-Legislation/France-to-allow-biosimilars-substitution (Accessed 11 May 2019).

[B19] GaBi (2017). Patent expiry dates for best-selling biologicals. Generics Biosimilars Initiat. J. 4, 178–179. 10.5639/gabij.2015.0404.040

[B20] GaBi (2018). France aims to reach 80% biosimilar penetration by 2022. Available at: http://www.gabionline.net/Policies-Legislation/France-aims-to-reach-80-biosimilar-penetration-by-2022 (Accessed May 2019).

[B21] Gleeson D. Townsend B. Lopert R. Lexchin J. Moir H. (2019). Financial costs associated with monopolies on biologic medicines in Australia. Aust. Health Rev. 43, 36–42. 10.1071/AH17031 29116927

[B22] Godman B. Allocati E. Moorkens E. (2019). Ever-Evolving landscape of biosimilars in Canada; findings and implications from a global perspective. GaBI Journal. 8(3), 93–97. 10.5639/gabij.2019.0803.012

[B23] Gulacsi L. Brodszky V. Baji P. Rencz F. Pentek M. (2017). The rituximab biosimilar CT-P10 in rheumatology and cancer: a budget impact analysis in 28 European countries. Adv. Ther. 34, 1128–1144. 10.1007/s12325-017-0522-y 28397080 PMC5427122

[B24] Hara F. Tajima K. Tanabe K. (2019). Current situation and challenges regarding biosimilars in Japan: an example of trastuzumab biosimilars for breast cancer. Future Oncol. 15 (12), 1353–1361. 10.2217/fon-2018-0957 30767568

[B25] Haustein R. de Millas C. Höer A. Häussler P. B. (2003). Saving money in the European healthcare systems with biosimilars. Generics Biosimilars Initiat. J. 1, 120–126. 10.5639/gabij.2012.0103-4.036

[B26] Health Improvement Scotland (NHS Scotland) (2015). Biosimilar medicines: a national prescribing framework (Scotland: Health Improvement Scotland (NHS Scotland)).

[B27] Health Improvement Scotland (NHS Scotland) (2018). Biosimilar medicines: a national prescribing framework (Scotland: Health Improvement Scotland (NHS Scotland)).

[B28] IQVIA (2009). IMS MIDAS user guide: IMS MIDAS quantum help. IQVIA. Accessed 22 Jul 2019.

[B29] Jorgensen K. K. Olsen I. C. Goll G. L. Lorentzen M. Bolstad N. Haavardsholm E. A. (2017). Switching from originator infliximab to biosimilar CT-P13 compared with maintained treatment with originator infliximab (NOR-SWITCH): a 52-week, randomised, double-blind, non-inferiority trial. Lancet 389, 2304–2316. 10.1016/S0140-6736(17)30068-5 28502609

[B30] Jung G. W. (2015). South Korean guidelines for biosimilars. GaBi J. 4 (2), 93–94. 10.5639/gabij.2015.0402.019

[B31] Kanavos P. Ferrario A. Vandoros S. Anderson G. F. (2013). Higher US branded drug prices and spending compared to other countries may stem partly from quick uptake of new drugs. Health Affairs 32 (4), 753–761. 10.1377/hlthaff.2012.0920 23569056

[B32] Komaki Y. Yamada A. Komaki F. Micic D. Ido A. Sakuraba A. (2017). Systematic review with meta-analysis: the efficacy and safety of CT-P13, a biosimilar of anti-tumour necrosis factor-alpha agent (infliximab), in inflammatory bowel diseases. Aliment Pharmacol. Ther. 45, 1043–1057. 10.1111/apt.13990 28239873

[B33] Kostic M. Djakovic L. Sujic R. Godman B. Jankovic S. M. (2017). Inflammatory Bowel Diseases (Crohn s Disease and Ulcerative Colitis): Cost of Treatment in Serbia and the Implications. Appl. Health Econom. Health Policy 15 (1), 85–93. 10.1007/s40258-016-0272-z PMC5253143 27587010

[B34] Kwon H. Y. Godman B. (2016). Do newly marketed generic medicines expand markets using descriptive time series analysis and mixed logit models? Korea as an exemplar and its implications. BMC Health Serv. Res. 16, 130. 10.1186/s12913-016-1356-z 27080530 PMC4832488

[B35] Lee H. Lee T. (2013). Impact of price control on drug expenditure and factors associated with the drug switch among statins: analysis of HIRA-NPS data. Kor. J. Health Policy Manage. 23, 112–123. 10.4332/KJHPA.2013.23.2.112

[B36] Leporowski A. Godman B. Kurdi A. MacBride-Stewart S. Ryan M. Hurding S. (2018). Ongoing activities to optimize the quality and efficiency of lipid-lowering agents in the Scottish national health service: influence and implications. Expert Rev. Pharmacoeconom. Outcomes Res. 18 (6), 655–666. 10.1080/14737167.2018.1501558 30014725

[B37] Lubloy A. (2014). Factors affecting the uptake of new medicines: a systematic literature review. BMC Health Serv. Res. 14, 469. 10.1186/1472-6963-14-469 25331607 PMC4283087

[B38] Magazzini L. Pammolli F. Riccaboni M. (2004). Dynamic competition in pharmaceuticals. Patent expiry, generic penetration, and industry structure. Eur. J. Health Econ. 5, 175–182. 10.1007/s10198-003-0218-x 15452754

[B39] Malik A. (2018) World preview 2018, outlook to 2024. EvaluatePharma, 11th ed. (EvaluatePharma). https://www.evaluate.com/thought-leadership/pharma/evaluatepharma-world-preview-2018 outlook-2024 (Accessed 13 Jan 2020).

[B40] Mestre-Ferrandiz J. Towse A. Berdud M. (2016). Biosimilars: how can payers get long-term savings? Pharmacoeconomics 34, 609–616. 10.1007/s40273-015-0380-x 26792791 PMC4863918

[B41] Ministère des Solidarités et de la Santé (2018). Instruction n° DGOS/PF2/DSS/1C/DGS/PP2/CNAMTS/2017/244 du 3 août 2017 relative aux médicaments biologiques, à leurs similaires ou « biosimilaires », et à l"interchangeabilité en cours des traitements. Ministère des Solidarités et de la Santé. Available at: http://solidarites-sante.gouv.fr/fichiers/bo/2017/17-10/ste_20170010_0000_0032.pdf (Accessed 22 Dec. 2019).

[B42] Ministère des Solidarités et de la Santé (2018). Instruction no DSS/1C/DGOS/PF2/2018/42 du 19 Février 2018 relative à l"incitation à la prescription hospitalière de médicaments biologiques similaires lorsqu"ils sont délivrés en ville. Available at: http://solidarites-sante.gouv.fr/fichiers/bo/2018/18-03/ste_20180003_0000_0090.pdf (Accessed 22 Dec. 2019).

[B43] Ministry of Government Legislation (2020a). Korean act on contracts to which the state is a party. Article 4. Available at: http://www.law.go.kr/lsInfoP.do?lsiSeq=199735&urlMode=engLsInfoR&viewCls=engLsInfoR#0000 (Accessed 23 Dec. 2019).

[B44] Ministry of Government Legislation (2020b). Korean Act on contracts to which a local government is a party: Article 5 (Ministry of Government Legislation). Available at: http://www.law.go.kr/lsInfoP.do?lsiSeq=200186&urlMode=engLsInfoR&viewCls=engLsInfoR#0000 (Accessed 23 Dec. 2019).

[B45] Ministry of Government Legislation (2020c). Korean act on the management of public institutions.:Article 44 (Ministry of Government Legislation). Available at: http://www.law.go.kr/lsInfoP.do?lsiSeq=188522&urlMode=engLsInfoR&viewCls=engLsInfoR#0000 (Accessed 23 Dec. 2019).

[B46] Ministry of Health and Welfare (2019). Korean biosimilar price calculation method (Ministry of Health and Welfare). http://www.law.go.kr/admRulSc.do?tabMenuId=tab107&query=약제liBgcolor12# (Accessed 22 May 2019).

[B47] Ministry of Health, Labour and Welfare (2018). NHI price calculation method. Available at: www.mhlw.go.jp/file/06-Seisakujouhou-12400000-Hokenkyoku/0000193793.pdf.

[B48] Moorkens E. Vulto A. G. Huys I. Dylst P. Godman B. Keuerleber S. (2017). Policies for biosimilar uptake in Europe: An overview. PloS One 12, e0190147. 10.1371/journal.pone.0190147 29284064 PMC5746224

[B49] Moorkens E. Simoens S. Troein P. Declerck P. Vulto A. G. Huys I. (2019). Different Policy Measures and Practices between Swedish Counties Influence Market Dynamics: Part 1-Biosimilar and Originator Infliximab in the Hospital Setting. BioDrugs : Clin. Immunotherapeut. Biopharmaceut. Gene Ther. 33 (3), 285–297. 10.1007/s40259-019-00345-6 PMC6533410 30945207

[B50] Mulcahy A. W. Hlávka J. P. Case S. R. (2017). Perspective:biosimilar cost savings in the United States (RAND corporation). Available at: https://www.rand.org/content/dam/rand/pubs/perspectives/PE200/PE264/RAND_PE264.pdf (Accessed 13 Jan 2020).

[B51] NHS England (2015). What is a Biosimilar Medicine?. [03/2020] Available from: https://www.gov.uk/government/publications/what-is-a-biosimilar-medicine (Accessed on March. 2020).

[B52] NHS England (2017a). Commissioning framework for biological medicines (including biosimilar medicines) (NHS England). Available at: https://www.england.nhs.uk/wp-content/uploads/2017/09/biosimilar-medicines-commissioning-framework.pdf (Accessed 13 Jan 2020).

[B53] NHS England (2017b). The Cancer Vanguard, [04/2020] Available from: https://cancervanguard.nhs.uk/biosimilars-getting-it-right-first-time/ (Accessed in April. 2020).

[B54] NHS England (2019a). What is a Biosimilar Medicine?. [03/2020] Available from: https://www.england.nhs.uk/publication/what-is-a-biosimilar-medicine/ (Accessed on March. 2020).

[B55] NHS England (2019b). Regional Medicines Optimisation Committees Operating Model. [04/2020] Available from: https://www.england.nhs.uk/publication/regional-medicines-optimisation-committee-operating-guidance-and-recruitment-information/ (Accessed April.2020).

[B56] NHS Scotland (2016). Effective prescribing programme-optimising the safe and effective use of biological medicines:case studies (NHS Scotland). Available at: http://www.healthcareimprovementscotland.org/our_work/technologies_and_medicines/adtc_resources/biosimilar_meds_case_studies.aspx (Accessed 13 Jan 2020).

[B57] NHS Scotland (2017). Secondary care national therapeutic indicators 2018/19 (NHS Scotland). Available at: https://www.therapeutics.scot.nhs.uk/wp-content/uploads/2018/08/Secondary-Care-National-Therapeutic-Indicators-Version-1.0.pdf (Accessed 13 Jan 2020).

[B58] OECD Health Database (2017). Health care resources: hospitals: public hospitals rate = number of public owned hospitals in Korea/number of hospitals in Korea (OECD). Available at: https://stats.oecd.org/index.aspx?DataSetCode=HEALTH_STAT (Accessed Dec 2019).

[B59] Ordre national des pharmaciens (2020). Biosimilaires: la loi de financement de la Sécurité sociale pour 2020 supprime le droit de substitution. [Biosimilars: the law on financing of the social security for 2020 abolished the right of substitution]. http://www.ordre.pharmacien.fr/Communications/Les-actualites/Biosimilaires-la-loi-de-financement-de-la-Securite-sociale-pour-2020-supprime-le-droit-de-substitution Accessed 8 May 2020.

[B60] Pivot X. Bondarenko I. Nowecki Z. Dvorkin M. Trishkina E. Ahn J. H. (2018). A phase III study comparing SB3 (a proposed trastuzumab biosimilar) and trastuzumab reference product in HER2-positive early breast cancer treated with neoadjuvant-adjuvant treatment: final safety, immunogenicity and survival results. Eur. J. Cancer 93, 19–27. 10.1016/j.ejca.2018.01.072 29448072

[B61] Putrik P. Ramiro S. Kvien T. K. Sokka T. Pavlova M. Uhlig T. (2014). Inequities in access to biologic and synthetic DMARDs across 46 European countries. Ann. Rheumatic Dis. 73 (1), 198–206. 10.1136/annrheumdis-2012-202603 23467636

[B62] Razanskaite V. Bettey M. Downey L. Wright J. Callaghan J. Rush M. (2017). Biosimilar infliximab in inflammatory bowel disease: outcomes of a managed switching programme. J. Crohns Colitis 11, 690–696. 10.1093/ecco-jcc/jjw216 28130330

[B63] Rémuzat C. Dorey J. Cristeau O. Ionescu D. Radiere G. Toumi M. (2017a). Key drivers for market penetration of biosimilars in Europe. J. Mark Access Health Policy 5 (1), 1272308. 10.1080/20016689.2016.1272308 28265349 PMC5328350

[B64] Rémuzat C. Kapusniak A. Caban A. Ionescu D. Radiere G. Mendoza C. (2017b). Supply-side and demand-side policies for biosimilars: an overview in 10 European member states. J. Mark Access Health Policy 5, 1307315. 10.1080/20016689.2017.1307315 28740617 PMC5508392

[B65] Richardson E. Kesselheim A. S. Paradise J. Borj K. Lott R. (2013). Health policy brief: biosimilars. Health Aff. 1–5.

[B66] Sagonowsky E. (2019). AbbVie’s massive Humira discounts are stifling Netherlands biosimilars: report. Fierce Pharma. https://www.fiercepharma.com/pharma/abbvie-stifling-humira-biosim-competition-massive-discounting-dutch-report (Accessed 13 Jan 2020).

[B67] Santos S. B. Sousa Lobo J. M. Silva A. C. (2019). Biosimilar medicines used for cancer therapy in Europe: a review. Drug Discovery Today 24, 293–299. 10.1016/j.drudis.2018.09.011 30244082

[B68] Schieber L. Z. Guy G. P. Jr. Seth P. Young R. Mattson C. L. Mikosz C. A. (2019). Trends and Patterns of Geographic Variation in Opioid Prescribing Practices by State, United States, 2006-2017. JAMA Netw. Open 2 (3), e190665. 10.1001/jamanetworkopen.2019.0665 30874783 PMC6484643

[B69] Simon-Kucher and Partners. Bonn (2016). Payers’ price & market access policies supporting a sustainable biosimilar medicines market.

[B70] Smeeding J. Malone D. C. Ramchandani M. Stolshek B. Green L. Schneider P. (2019). Biosimilars: considerations for payers. P. T. 44, 54–63. 30766011 PMC6355057

[B71] Stebbing J. Baranau Y. V. Baryash V. Manikhas A. Moiseyenko V. Dzagnidze G. (2017). Double-blind, randomized phase III study to compare the efficacy and safety of CT-P6, trastuzumab biosimilar candidate versus trastuzumab as neoadjuvant treatment in HER2 positive early breast cancer (EBC). J. Clin. Oncol. 35, 510. 10.1200/JCO.2017.35.15_suppl.510

[B72] Syrop J. (2017). NHS May use more “stick” than “carrot” to ensure prescribing of cheaper biosimilars. (AJMC The center for Biosimilars). Available at: https://www.centerforbiosimilars.com/news/nhs-may-use-more-stick-than-carrot-to-ensure-prescribing-of-cheaper-biosimilars.

[B73] Tanabe Y. Sugimoto N. Muto M. F. (2016). Field survey on the recognition and intention of using follow-on biologics (biosimilars) in physicians and pharmacists. Prog. Med. 36, 291–300.

[B74] Thill M. (2019). Biosimilar trastuzumab in clinical trials: differences or not? Breast Care (Basel) 14, 17–22. 10.1159/000496503 31019438 PMC6465702

[B75] TLV (2018). International price comparison of pharmaceuticals 2017 (Stockholm: TLV).

[B76] US Food and Drug Administration (2019). Information on biosimilars. US Food and Drug Administration. Available at: https://www.fda.gov/Drugs/DevelopmentApprovalProcess/HowDrugsareDevelopedandApproved/ApprovalApplications/TherapeuticBiologicApplications/Biosimilars/%0A%0A (Accessed 10 May 2019).

[B77] Van Boeckel T. P. Gandra S. Ashok A. Caudron Q. Grenfell B. T. Levin S. A. (2014). Global antibiotic consumption 2000 to 2010: an analysis of national pharmaceutical sales data. Lancet Infect. Dis. 14, 742–750. 10.1016/S1473-3099(14)70780-7 25022435

[B78] Vergara-Dangond C. Saez Bello M. Climente Marti M. Llopis Salvia P. Alegre-Sancho J. J. (2017). Effectiveness and safety of switching from innovator infliximab to biosimilar CT-P13 in inflammatory rheumatic diseases: a real-world case study. Drugs R. D. 17, 481–485. 10.1007/s40268-017-0194-8 28667384 PMC5629134

[B79] Vogler S. Schneider P. (2017). Do pricing and usage-enhancing policies differ between biosimilars and generics? Findings from an international survey. Generics Biosimilars Initiat. J. 6, 79–88. 10.5639/gabij.2017.0602.015

[B80] WHO (2019). WHO ATC/DDD index (WHO). Available at: https://www.whocc.no/atc_ddd_index/?code=L04AB02 (Accessed 13 Jan 2020).

[B81] World Health Organization (2018). Technical report: pricing of cancer medicines and its impacts: a comprehensive technical report for the world health assembly (Geneva, Switzerland: World Health Organization).

[B82] Yumpto T. (2017). Update of Drug Pricing System in Japan, presentation file (Ministry of Health, Labor and Welfare. https://www.pmda.go.jp/files/000226220.pdf (Accessed on July 5, 2020).

